# Supplementary material for: SPRING licenses S1P-mediated cleavage of SREBP2 by displacing an inhibitory pro-domain
Source: Nat Commun. 2024 Jul 9;15:5732. doi: 10.1038/s41467-024-50068-8 (PMC11231238; doi:10.1038/s41467-024-50068-8)
Supplement: Supplementary file 1 — Supplementary Information [file 41467_2024_50068_MOESM1_ESM.pdf]

Supplementary Information:

SPRING licenses S1P-mediated cleavage of SREBP2 by displacing an inhibitory pro-domain

Sebastian Hendrix[1], Vincent Dartigue[2], Hailee Hall[2], Shrankhla Bawaria[2], Jenina Kingma[1], Bilkish Bajaj[2], Noam Zelcer[1]*, and Daniel L Kober[2]*

[1]Department of Medical Biochemistry, Amsterdam UMC, Amsterdam Cardiovascular Sciences and Gastroenterology and Metabolism, University of Amsterdam, Meibergdreef 9, 1105AZ, Amsterdam, the Netherlands

[2]Department of Biochemistry, The University of Texas Southwestern Medical Center, Dallas, TX 75390, USA

* Corresponding Authors: daniel.kober@utsouthwestern.edu and n.zelcer@amsterdamumc.nl

Supplementary Figure 1

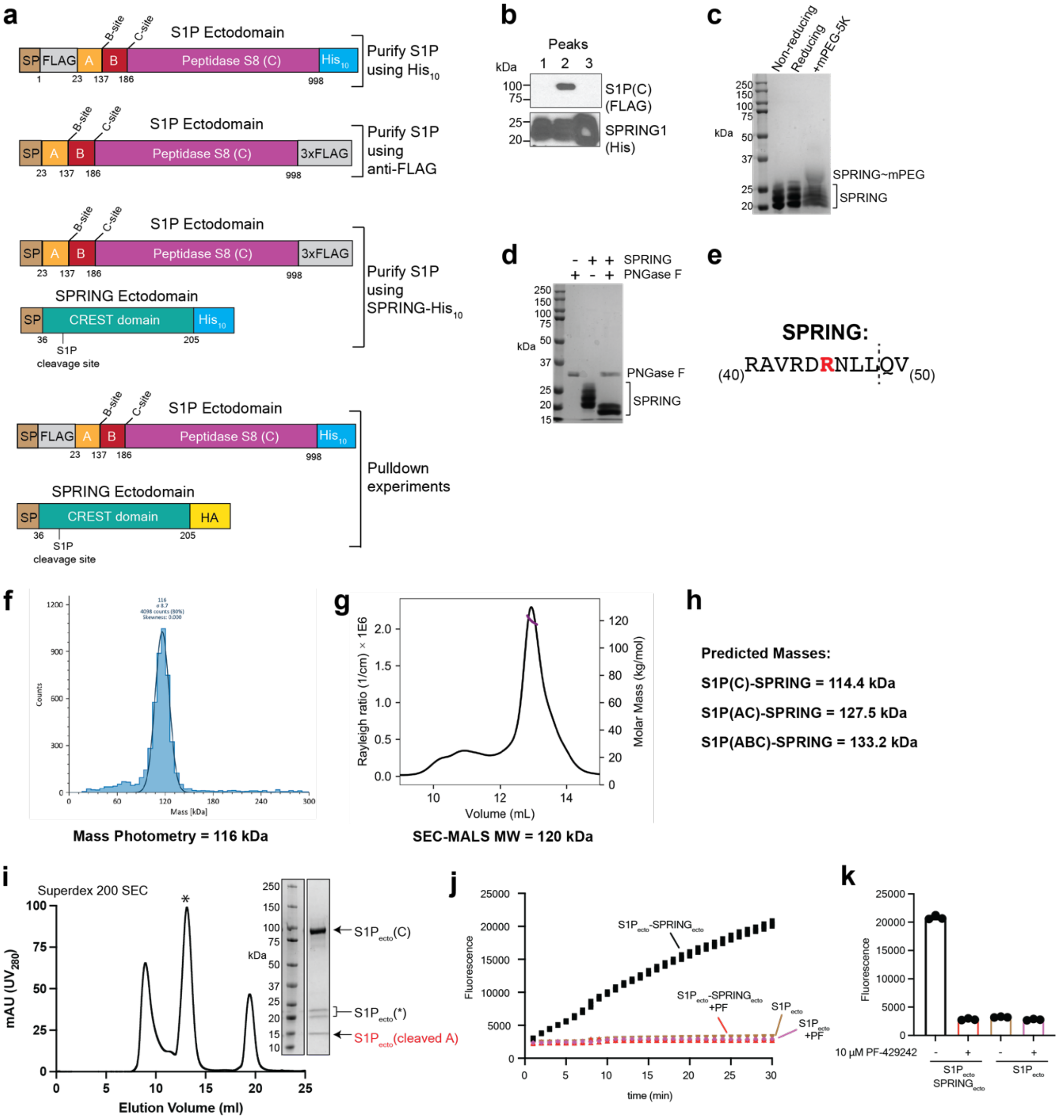

Supplementary Figure 1.

a) Schematic for protein expression constructs used for biochemical and structural assays in this study.
b) Immunoblot analysis of peaks from **Fig. 1c**. $S1P_{ecto}$ is detected using anti-FLAG antibodies and $SPRING_{ecto}$ is detected using anti-His antibodies.
c) $SPRING_{ecto}$ from Fig. 1c peak 3 analyzed by SDS-PAGE in non-reducing or reducing conditions or else treated with 5 mM mPEG-5K to label unpaired cysteines.
d) Coomassie-stained SDS-PAGE analysis for N-linked glycosylation using PNGaseF using $SPRING_{ecto}$ from **Fig. 1c** Peak 3.
e) S1P cleavage sequence in SPRING.
f) Analysis of $S1P_{ecto}$-$SPRING_{ecto}$ complex using Mass Photometry.
g) Analysis of $S1P_{ecto}$-$SPRING_{ecto}$ complex using SEC-MALS. Black trace and left axis show scattering signal and the purple line and right axis show the calculated molecular weight of the SPRING-S1P complex.
h) Predicted masses for possible versions of the $S1P_{ecto}$-$SPRING_{ecto}$ complex containing different combinations of the pro-domains.
i) Gel filtration and Coomassie-stained SDS-PAGE analysis of $S1P_{ecto}$-3xFLAG purified using M2 anti-FLAG affinity resin.
j) Real-time SREBP2 proteolysis data measuring the activity of 10 nM $S1P_{ecto}$-3xFLAG or $S1P_{ecto}$-3xFLAG/$SPRING_{ecto}$-His10 complex cleaving 150 µM SREBP2 peptide in reactions containing 10 µM PF-429242 or DMSO vehicle control as indicated. Assay was measured using three technical replicates (n=3). Error bars are standard error of the mean. Representative of two independent experiments.
k) Endpoint data from (j). Assay was conducted with three technical replicates (n=3). Error bars are standard error of the mean. Representative of two independent experiments.

Uncropped immunoblots and SDS-PAGE gels are provided in the Source Data file.

Supplementary Figure 2

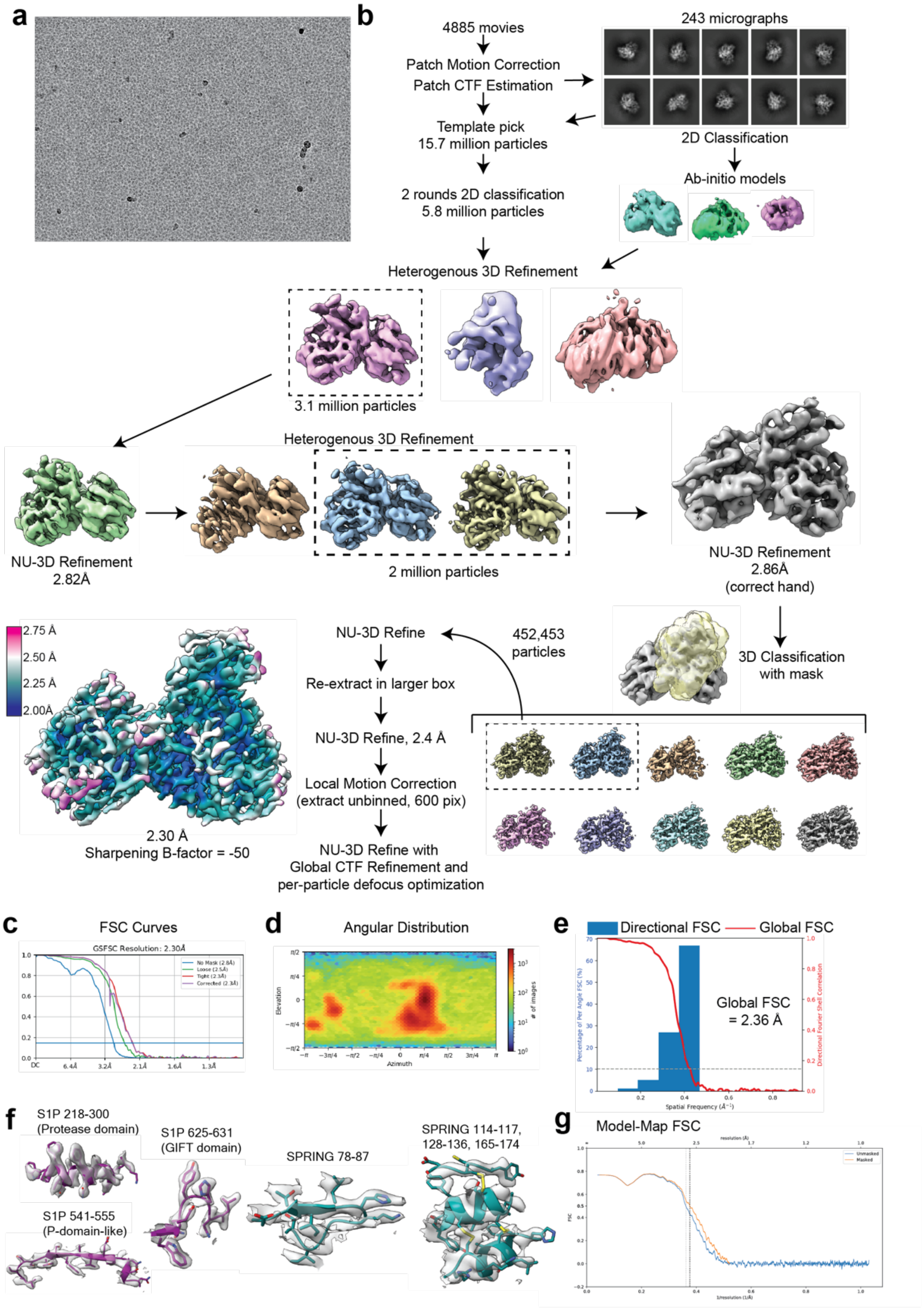

Supplementary Figure 2. Cryo-EM data processing of S1P$_{ecto}$-SPRING$_{ecto}$ complex.

a) Representative cryo-EM micrograph.
b) Flow-chart for data processing of S1P$_{ecto}$-SPRING$_{ecto}$ complexes, for details see *Methods*.
c) FSC curves.
d) Angular distribution plot.
e) Three-dimensional FSC plot.
f) Representative cryo-EM densities for the indicated segments of S1P$_{ecto}$ and SPRING$_{ecto}$. S1P$_{ecto}$ residues are colored magenta, SPRING$_{ecto}$ residues are colored teal.
g) Model-Map FSC curves.

Supplementary Figure 3

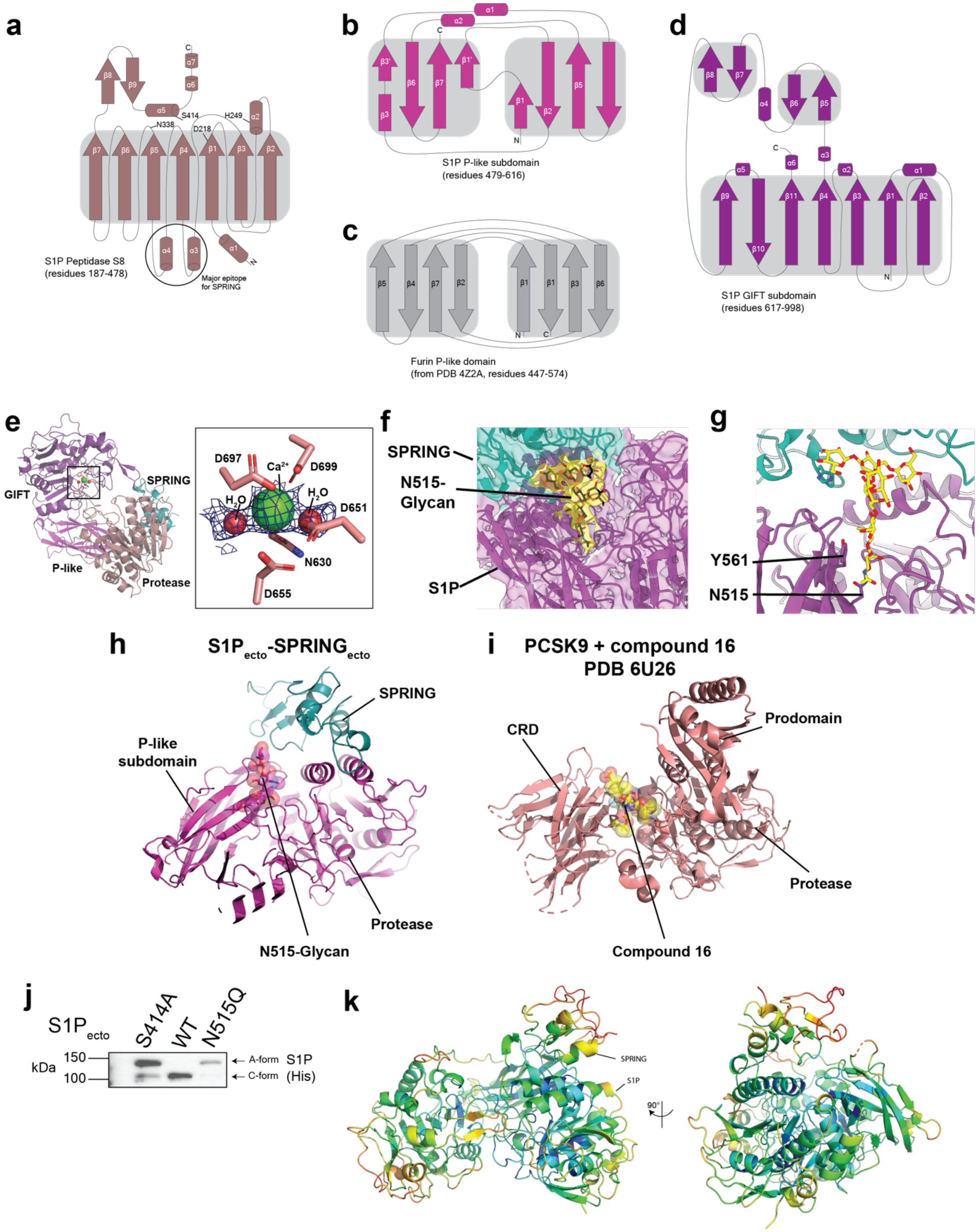

Supplementary Figure 3. Features of the $S1P_{ecto}$-$SPRING_{ecto}$ interaction.

a) Secondary structure topology of the S1P Peptidase S8 subdomain. Arrows indicate beta strands and cylinders indicate alpha helices. Beta sheets are indicated with grey background. N and C indicate termini.
b) Secondary structure topology of the S1P P-like subdomain. Arrows indicate beta strands and cylinders indicate alpha helices. Beta sheets are indicated with grey background. N and C indicate termini.
c) Secondary structure topology of the P domain from Furin based on PDB 4Z2A. Arrows indicate beta strands and cylinders indicate alpha helices. Beta sheets are indicated with grey background. N and C indicate termini.
d) Secondary structure topology of the S1P GIFT subdomain. Arrows indicate beta strands and cylinders indicate alpha helices. Beta sheets are indicated with grey background. N and C indicate termini.
e) Putative calcium site in the S1P GIFT subdomain. Left: Overall structure of $S1P_{ecto}$-$SPRING_{ecto}$ with SPRING colored teal, S1P colored magenta, and putative calcium molecule depicted as green sphere. Right: Closeup of putative calcium molecule and interacting residues of S1P.
f) Unsharpened map countered to level 0.117 in ChimeraX showing density for the N-linked glycan on $S1P_{ecto}^{N515}$. $S1P_{ecto}$ map and model are colored magenta, $SPRING_{ecto}$ map and model are teal, glycan map and sugars are yellow.
g) Atomic model of $S1P_{ecto}$ N515-glycan and interacting residues.
h) $S1P_{ecto}$-$SPRING_{ecto}$ model sliced to view the N515 glycan as if from the GIFT subdomain. The $S1P_{ecto}$ domains are shown as magenta cartoon and $SPRING_{ecto}$ is shown as teal cartoon. The N515-glycan is depicted as sticks inside transparent spheres.
i) PCSK9 with bound compound 16 (PDB 6U26) is shown as a cartoon with the compound depicted as sticks inside transparent spheres. Subdomains are labeled. CRD = Cys Rich Domain.
j) $S1P_{ecto}$ proteins secreted from transfected HEK293T cells analyzed by anti-His immunoblot.
k) Structure of S1Pecto-SPRINGecto colored by B-factor (Ca).

Uncropped immunoblots are provided in the Source Data file.

Supplementary Figure 4

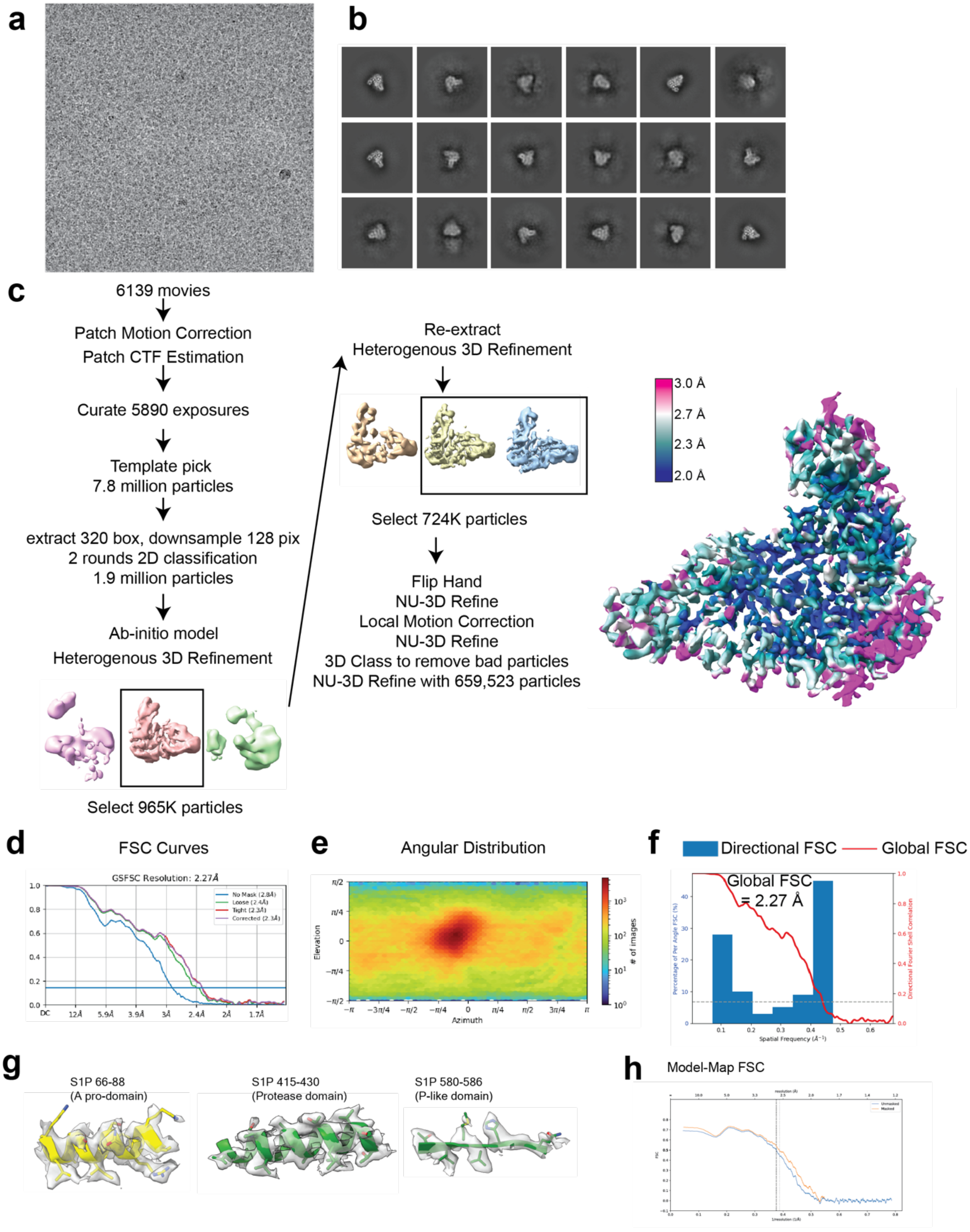

Supplementary Figure 4. Cryo-EM data processing of $S1P_{ecto}$.

a) Representative cryo-EM micrograph.
b) Selected 2D classes from picked particles.
c) Flow-chart for data processing of $S1P_{ecto}$, see *Methods* for details.
d) FSC curves.
e) Angular distribution plot.
f) Three-dimensional FSC plot.
g) Representative cryo-EM densities for the indicated segments of $S1P_{ecto}$. $S1P_{ecto}$ residues from the C-domain are colored green and $S1P_{ecto}$ residues from the A domain are colored yellow.
h) Model-Map FSC curves.

Supplementary Figure 5

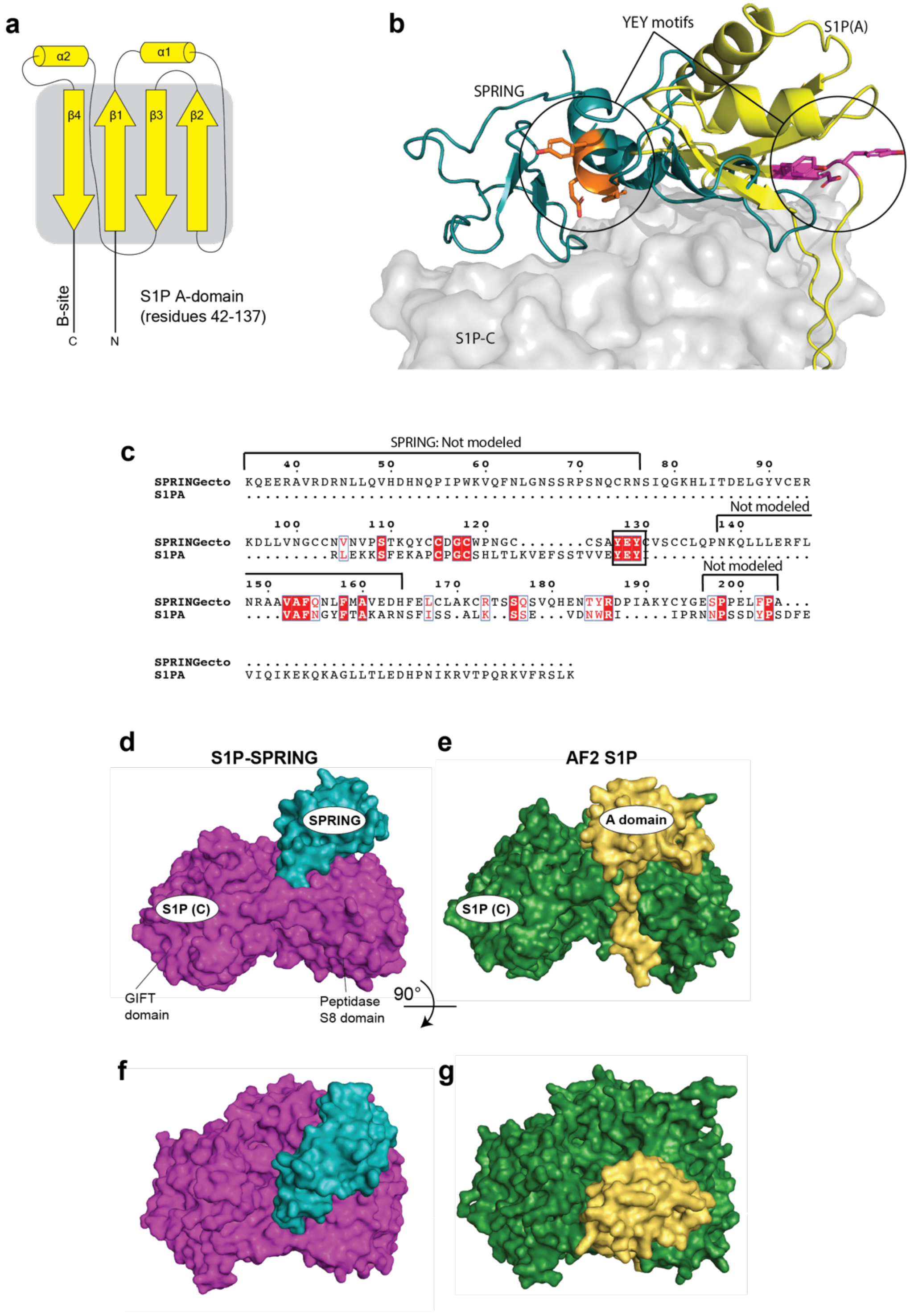

Supplementary Figure 5. $SPRING_{ecto}$ and the $S1P_{ecto}$ A-domain binding sites overlap.

a) Secondary structure topology of the S1P A-domain. Arrows indicate beta strands and cylinders indicate alpha helices. Beta sheets are indicated with grey background. N and C indicate termini.

b) SPRING and the A-domain bind the same epitope on S1P-C despite lacking structural similarity. The Peptidase S8 and P-like subdomains of $S1P_{ecto}$ are depicted in grey surface. SPRING and the A-domain are shown as cartoons. $SPRING_{ecto}$ is colored teal and the A-domain from $S1P_{ecto}$ is colored yellow. SPRING was placed by superposing the Peptidase S8 domains of the two forms of S1P. Two YEY motifs are shown as orange and magenta sticks and labeled with circles. Figure generated using Pymol.

c) Sequence alignment of SPRING and the S1P A-domain generated using Clustal and visualized using the ESPript 3 server. Residues of $SPRING_{ecto}$ that are not modeled in the cryo-EM structure are labeled with brackets. Residue numbers are based on SPRING. Red boxes with white letters highlight identical residues, white boxes with red letters indicate residues with similar physiochemical properties.

d) $S1P_{ecto}$-$SPRING_{ecto}$ structure determined using cryo-EM. $S1P_{ecto}$ is depicted as magenta surface. $SPRING_{ecto}$ is depicted as teal surface. $S1P_{ecto}$ subdomains are labeled.

e) The AF2 prediction for S1P is superposed to the $S1P_{ecto}$-$SPRING_{ecto}$ shown in (d). The C-domain is depicted as green surface. A-domain as yellow surface. The B-domain is predicted to be disordered and is not shown. Predicted structures of the signal peptide, linker regions, and C-terminal transmembrane helix are likewise omitted for clarity.

f) $S1P_{ecto}$-$SPRING_{ecto}$ depicted as in (d) and rotated 90° downwards towards the page.

g) $S1P_{ecto}$ depicted as in (e) and rotated 90° downwards towards the page.

Supplementary Figure 6

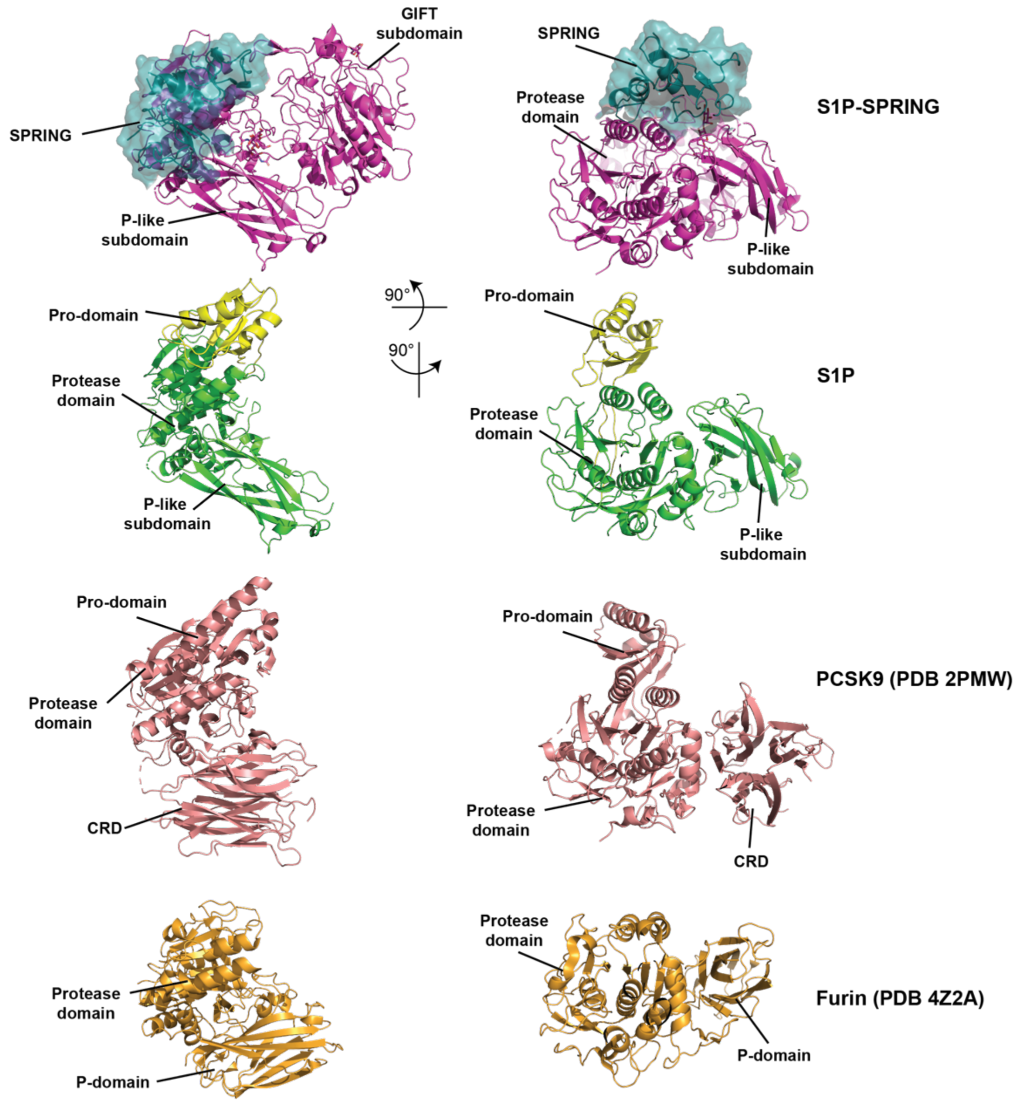

Supplementary Figure 6. Comparing S1P$_{ecto}$ and S1P$_{ecto}$-SPRING$_{ecto}$ to crystal structures of PCSK9 and Furin.

S1P$_{ecto}$-SPRING$_{ecto}$ (this study), S1P$_{ecto}$ (this study), PCSK9 (PDB 2PMW) and Furin (PDB 4Z2A) were superposed using their Peptidase domains. SPRING is shown with transparent teal surface and cartoon. S1P and other proteases shown with cartoons. All four proteins shown from two views to highlight changes in the orientations of the P-like subdomain (or equivalent subdomains), *right*; or else the clash of the pro-domain with SPRING, *left*.

Supplementary Table 1

**SPRING$_{ecto}$ mutagenesis primers**	
SPRING R45A	/5Phos/GCAGGAGGAGAGGGCAGTGAGAGATgcGAATCTCCTCCAGGTTCATGACC
SPRING R95A	/5Phos/CGGATGAACTCGGCTACGTTTGCGAGgcGAAGGATTTGCTGGTAAATGGCTGC
SPRING R174A	/5Phos/CTTTGAGTTGTGCCTGGCCAAATGCgcGACCTCATCTCAGAGCGTGCAGC
SPRING V180A	/5Phos/CCAAATGCAGGACCTCATCTCAGAGCgcGCAGCATGAGAACACCTACCGGGACCCC
**S1P$_{ecto}$ mutagenesis primers**	
S1P P317A	/5Phos/GGCGGCCCGGACTTCATGGATCATgcGTTTGTTGACAAGGTGTGGG
S1P N328A	/5Phos/GTTGACAAGGTGTGGGAATTAACAGCTgcCAATGTAATCATGGTTTCTGCTATTGGC
S1P N515Q	/5Phos/GGAGGAATGCCGACAGTTGTTcagGTCACCATCCTCAACGGCATGGG
S1P W556A	/5Phos/GTTGCCTTCTCCTACTCCTCGGTCTTAgcGCCTTGGTCGGGCTACCTGGCC
S1P R130/134E	/5Phos/CAAACGGGTCACGCCCCAAgaaAAAGTCTTTgaaTCCCTCAAGTATGCTGAATCTGACC

**S1P$_{FL}$ mutagenesis primers**	
S1P P317A fw	CTTCATGGATCATGCGTTTGTTGACAAG
S1P P317A rv	CTTGTCAACAAACGCATGATCCATGAAG
S1P N328A fw	GGAATTAACAGCTGCCAATGTAATCATGG
S1P N328A rv	CCATGATTACATTGGCAGCTGTTAATTCC
S1P W556A fw	TACTCCTCGGTCTTAGCGCCTTGGTCGGGCTA
S1P W556A rv	TAGCCCGACCAAGGCGCTAAGACCGAGGAGTA
S1P N515Q fw	GAATGCCGACAGTTGTTCAGGTCACCATCCTCAACG
S1P N515Q rv	CGTTGAGGATGGTGACCTGAACAACTGTCGGCATTC
**SPRING$_{FL}$ mutagenesis primers**	
SPRING R95A fw	CTACGTTTGCGAGGCGAAGGATTTGCTG
SPRING R95A rv	CAGCAAATCCTTCGCCTCGCAAACGTAG
Spring R174A fw	CTGGCCAAATGCGCGACCTCATCTCAG
Spring R174A rv	CTGAGATGAGGTCGCGCATTTGGCCAG
SPRING V180A fw	CATCTCAGAGCGCGCAGCATGAGAAC
SPRING V180A rv	GTTCTCATGCTGCGCGCTCTGAGATG
