# Supplementary material for: SPRING licenses S1P-mediated cleavage of SREBP2 by displacing an inhibitory pro-domain
Source: Nat Commun. 2024 Jul 9;15:5732. doi: 10.1038/s41467-024-50068-8 (PMC11231238; doi:10.1038/s41467-024-50068-8)
Supplement: Supplementary file 3 — Reporting Summary [file 41467_2024_50068_MOESM3_ESM.pdf]

nature portfolio

Corresponding author(s): Kober, Daniel
Zelcer, Noam

Last updated by author(s): Jun 23, 2024

Reporting Summary

Nature Portfolio wishes to improve the reproducibility of the work that we publish. This form provides structure for consistency and transparency in reporting. For further information on Nature Portfolio policies, see our Editorial Policies and the Editorial Policy Checklist.

Statistics

For all statistical analyses, confirm that the following items are present in the figure legend, table legend, main text, or Methods section.

n/a	Confirmed	
☐	☒	The exact sample size (*n*) for each experimental group/condition, given as a discrete number and unit of measurement
☐	☒	A statement on whether measurements were taken from distinct samples or whether the same sample was measured repeatedly
☐	☒	The statistical test(s) used AND whether they are one- or two-sided<br>*Only common tests should be described solely by name; describe more complex techniques in the Methods section.*
☐	☒	A description of all covariates tested
☐	☒	A description of any assumptions or corrections, such as tests of normality and adjustment for multiple comparisons
☐	☒	A full description of the statistical parameters including central tendency (e.g. means) or other basic estimates (e.g. regression coefficient) AND variation (e.g. standard deviation) or associated estimates of uncertainty (e.g. confidence intervals)
☐	☒	For null hypothesis testing, the test statistic (e.g. *F*, *t*, *r*) with confidence intervals, effect sizes, degrees of freedom and *P* value noted<br>*Give P values as exact values whenever suitable.*
☒	☐	For Bayesian analysis, information on the choice of priors and Markov chain Monte Carlo settings
☒	☐	For hierarchical and complex designs, identification of the appropriate level for tests and full reporting of outcomes
☒	☐	Estimates of effect sizes (e.g. Cohen's *d*, Pearson's *r*), indicating how they were calculated

Our web collection on statistics for biologists contains articles on many of the points above.

Software and code

Policy information about availability of computer code

Data collection	SerialEM software was used to collect cryo-EM data
Data analysis	Prism v10.1.0 software was used for statistical analyses of western blot experiments.<br>CryoSparc v4.2 was used to analyze cryo-EM data.<br>Coot, ISOLDE, and Phenix were used to build and refine the atomic models.<br>ChimeraX and Pymol were used to analyze and visualize cryo-EM maps and atomic models.

For manuscripts utilizing custom algorithms or software that are central to the research but not yet described in published literature, software must be made available to editors and reviewers. We strongly encourage code deposition in a community repository (e.g. GitHub). See the Nature Portfolio guidelines for submitting code & software for further information.

Data

Policy information about availability of data

All manuscripts must include a data availability statement. This statement should provide the following information, where applicable:

- Accession codes, unique identifiers, or web links for publicly available datasets
- A description of any restrictions on data availability
- For clinical datasets or third party data, please ensure that the statement adheres to our policy

The atomic models are deposited in the Protein Data Bank (PDB) with codes 8UW8 (S1Pecto-SPRINGecto) and 8UWC (S1Pecto) and the cryo-EM maps are deposited in the Electron Microscopy Data Bank (EMDB) with the accession codes: 42639 (S1Pecto-SPRINGecto) and 42661 (S1Pecto).

Research involving human participants, their data, or biological material

Policy information about studies with human participants or human data. See also policy information about sex, gender (identity/presentation), and sexual orientation and race, ethnicity and racism.

Reporting on sex and gender	NA
Reporting on race, ethnicity, or other socially relevant groupings	NA
Population characteristics	NA
Recruitment	NA
Ethics oversight	NA

Note that full information on the approval of the study protocol must also be provided in the manuscript.

Field-specific reporting

Please select the one below that is the best fit for your research. If you are not sure, read the appropriate sections before making your selection.

[x] Life sciences [ ] Behavioural & social sciences [ ] Ecological, evolutionary & environmental sciences

For a reference copy of the document with all sections, see nature.com/documents/nr-reporting-summary-flat.pdf

Life sciences study design

All studies must disclose on these points even when the disclosure is negative.

Sample size	No sample size calculations were conducted. For western blot data, statistical significance was tested using one-way ANOVA with Holm-Sidak post hoc analysis.
Data exclusions	No data were excluded.
Replication	All experiments were reproduced on at least three independent experiments.
Randomization	NA
Blinding	Experiments were not blinded. These biochemical experiments rely on the included experimental controls.

Reporting for specific materials, systems and methods

We require information from authors about some types of materials, experimental systems and methods used in many studies. Here, indicate whether each material, system or method listed is relevant to your study. If you are not sure if a list item applies to your research, read the appropriate section before selecting a response.

Materials & experimental systems

n/a	Involved in the study	
	x	Antibodies
	x	Eukaryotic cell lines
x		Palaeontology and archaeology
x		Animals and other organisms
x		Clinical data
x		Dual use research of concern
x		Plants

Methods

n/a	Involved in the study	
x		ChIP-seq
x		Flow cytometry
x		MRI-based neuroimaging

Antibodies

Antibodies used	The following antibodies and dilutions were used in this study: anti-FLAG M2 clone (Sigma-Aldrich Cat # F1804, diluted 1:1000), HIS.H8 anti-His antibody (Sigma-Aldrich Cat # 05-949, diluted 1:1000), HA Tag Monoclonal Antibody (2-2.2.14) (ThermoScientific Cat # 26183, diluted 1:1000), V5-tag (Invitrogen Cat# R960-25, diluted 1:1000), anti-alpha-Tubulin (Sigma Cat# T9026, diluted 1:2000), HRP-conjugated anti-Myc tag antibody (Thermo Scientific Cat# R951-25, diluted 1:1000) and Myc-tag (Cell Signaling Cat # 2276S, diluted 1:1000).
Validation	These well-validated antibodies are specific for their respective epitope tags. In our work, this was confirmed using mock-transfected wells where empty plasmids were used as expression controls. Further validation data are available from the manufacturer's websites.

Eukaryotic cell lines

Policy information about cell lines and Sex and Gender in Research

Cell line source(s)	HEK293T cells were obtained from ATCC (ATCC CRL-3216)<br>HEK293S GnTI- cells were obtained from ATCC (CRL-3022).
Authentication	Cell lines were not validated further.
Mycoplasma contamination	Cells were confirmed to be free of mycoplasma using InVivogen MycoStrip kit.
Commonly misidentified lines (See ICLAC register)	NA

Plants

Seed stocks	NA
Novel plant genotypes	NA
Authentication	NA
